# Comparable outcomes between quadriceps and hamstring tendon autografts for isolated medial patellofemoral ligament reconstruction: A systematic review of studies with minimum 2‐year follow‐up

**DOI:** 10.1002/jeo2.70513

**Published:** 2025-12-01

**Authors:** Ehson Maleki, Ali Ahmadi Pirshahid, Lee R. Benaroch, Raheef Alatassi, Hussein Maleki, Debra Bartley, Robert Litchfield, Patrick Thornley, Karthick Rangasamy

**Affiliations:** ^1^ Baylor College of Medicine School of Medicine Houston Texas USA; ^2^ Division of Orthopaedic Surgery, Department of Surgery Western University London Ontario Canada; ^3^ College of Natural Sciences and Mathematics University of Houston Houston Texas USA; ^4^ Department of Orthopaedic Surgery Post Graduate Institute of Medical Education and Research (PGIMER) Chandigarh India

**Keywords:** hamstring, knee, medial patellofemoral ligament reconstruction (MPFL), patellar dislocation, patellar instability, quadriceps

## Abstract

**Purpose:**

To systematically review existing evidence to discern the most effective autograft between the hamstring tendon (HT) and the quadriceps tendon (QT) for isolated medial patellofemoral ligament (MPFL) reconstruction to address recurrent patellar instability.

**Methods:**

Medline, Embase and Scopus search strategies were developed following the preferred reporting items for systematic reviews and meta‐analyses (PRISMA) guidelines. Only studies that included isolated MPFL reconstruction (MPFLR) using quadriceps or hamstring autograft on patients 12 years or older affected by recurrent patellofemoral instability with a minimum follow‐up of 2 years were considered. The primary outcome of this review was the re‐dislocation rate, and secondary outcomes included patient‐reported outcome measures (PROMs), complications and radiographic parameters.

**Results:**

Twelve studies, comprising 339 patients who underwent isolated MPFLR using HT autograft and 139 patients using QT autograft (follow‐up range, 24–120 months), were included. Only one HT study reported patellar re‐dislocations following MPFLR, with four patients requiring revision surgery. All other studies reported nil patellar re‐dislocation. All studies showed significant mean improvements in postoperative Kujala (range of improvement in HT group from 26.5 to 41.4; QT group from 6.6 to 53.0) and Lysholm scores (range of improvement in HT group from 41.7 to 45.84; QT group from 10.9 to 46.0). Five studies provided postoperative Tegner Activity scores (postoperative range: 4.0–7.6). Four studies reported significant decreases in patellar tilt angle (PTA) postoperatively (range in HT group: 12.6–16.3; QT group: 13.0). Five studies reported significant decreases in the pain scale (visual analogue scale) postoperatively (range: 1.0–1.9). Seven studies reported that all patients had a full range of motion postoperatively.

**Conclusion:**

There is no significant difference in re‐dislocation rate or PROMs between isolated MPFLR done with HT compared to QT autograft. Major limitations of this systematic review include a lack of strong outcome measures specific to patients with patellofemoral instability, along with few comparative studies.

**Level of Evidence:**

Level IV, a systematic review of Level I–IV studies.

**Abbreviations**
HT: hamstring tendon
MINORS: methodological index for nonrandomised studies
MPFL: medial patellofemoral ligament
MPFLR: MPFL reconstruction
PRISMA: preferred reporting items for systematic reviews and meta‐analyses
PTA: patellar tilt angle
QT: quadriceps tendon
ROM: range of motion
TT‐TG: tibial tuberosity‐trochlear groove
VAS: visual analogue scale for pain

## INTRODUCTION

Lateral patellar dislocation is a common cause of patellofemoral instability, particularly in adolescents and young adults. While there are numerous demographic and anatomic risk factors for this pathology, the first incidence of patellar dislocation puts the patient at a 20%–30% risk of repeat dislocation [10]. Most patients with recurrent patellar instability have been reported to have a disrupted medial patellofemoral ligament (MPFL) [12]. The MPFL is the main static restraint to lateral dislocation of the patella in early flexion (0°–30°), assisting the patella in engaging the trochlear groove, after which bony anatomy becomes the primary restraint to lateral dislocation [9, 34]. Naturally, cases of recurrent patellar instability are commonly treated with reconstruction of the MPFL, either in isolation or concurrent with other procedures aiming to correct knee alignment or anatomic risk factors such as trochlear dysplasia, patella alta, laterally located tibial tubercle and femorotibial malrotation [4].

MPFL reconstruction has been reported with auto‐ and allografts, as well as synthetic substitutes. Most commonly, an autologous hamstring tendon (HT) (semitendinosus or gracilis) has been used to reconstruct the MPFL, with good short‐to‐medium results reported [34]. This procedure involves harvesting the HT as a free graft, with subsequent attachment to the femoral origin (‘Schottle's point’) and insertion onto the superomedial portion of the patella. The use of quadriceps tendon (QT) autograft as a free graft was first described in 1997, with further modification by Steensen et al. which maintained the soft tissue attachment on the patellar side. This precludes the need for drilling the patella and avoids the potential complication of a fractured patella or implant breakage on the patellar side [31].

The goal of this systematic review was to provide a collection of studies that described the outcomes of isolated MPFL reconstruction using hamstring or quadriceps autograft in adults and adolescents older than 12 years. There have been numerous studies that have described outcomes of isolated MPFL reconstruction using either hamstring or quadriceps autograft. However, there remains a paucity of literature directly comparing the two commonly used autografts, with only two such papers available in the literature [23, 24]. The primary outcome of this review was re‐dislocation events, with secondary outcomes including functional scores, range of motion (ROM), complication rates, return to sport and radiographic parameters. We hypothesised that QT autografts would yield superior outcomes and fewer complications than HT autografts in isolated MPFL reconstruction.

## METHODS

### Eligibility criteria

The study protocol for this systematic review was registered prospectively on PROSPERO. This systematic review investigated the outcomes of MPFL reconstruction using QT or hamstring tendon (HT) autograft in patients aged 12 years or older affected by recurrent patellofemoral instability and unresponsive to conservative treatment. We included studies involving patients older than 12, where the femoral tunnel can be safely performed without significant risk of growth disturbance [6]. Only studies that included isolated MPFL reconstruction using QT or HT autograft with a minimum follow‐up of 2 years were considered. Studies that included patients with both MPFL reconstruction and other bony procedures, such as tibial tuberosity transfer or valgus corrective osteotomy cases, were excluded. Studies that used allografts or other synthetic grafts were excluded. Studies that included patients with a tibial tuberosity‐trochlear groove (TT‐TG) distance of >20 mm or trochlear dysplasia greater than Dejour B were excluded. Case series with fewer than 10 cases, case reports, nonclinical studies and studies with less than 2 years of minimum follow‐up were also excluded. Only articles published in English after the year 1997 were included, as it was the first year a partial‐thickness QT was used in MPFL reconstruction [2]. Patellofemoral re‐dislocation rate was the primary outcome in this review. Secondary outcomes included functional scores (visual analogue scale [VAS], Kujala and Lysholm scores), ROM, return to sport, complication profiles and radiographic parameters.

### Search strategy

**Figure 1:**
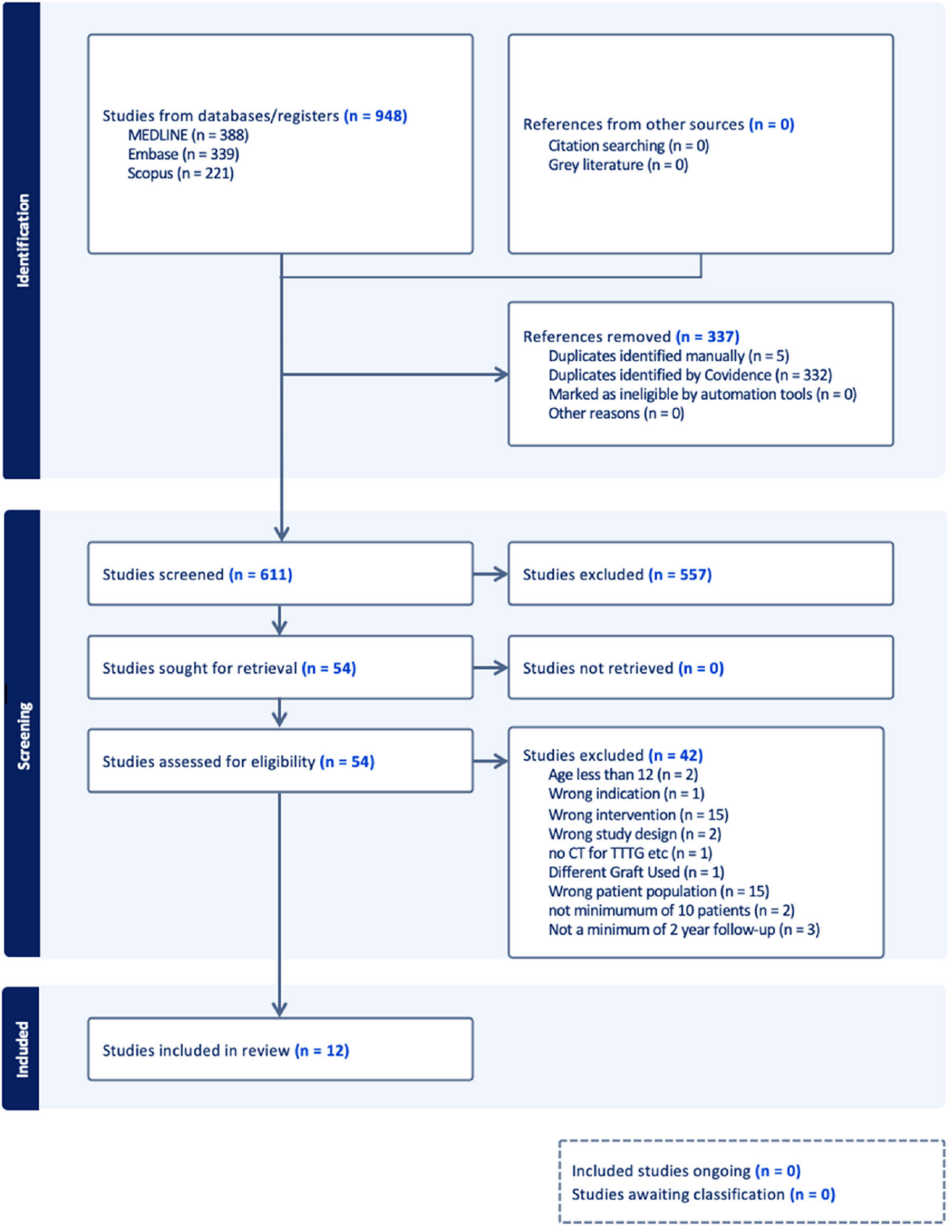
Preferred reporting items for systematic reviews and meta‐analyses flowchart.

This systematic review was conducted according to the 2020 PRISMA guidelines (Figure 1) [21]. One author performed the literature search in MEDLINE and Embase up to 28 February 2024. A second author conducted the literature search in Scopus until 3 March 2024. The database search string used was ([Quadriceps] OR [Hamstring] OR [Gracilis] OR [Semitendinosus]) AND ([MPFL] OR [Medial AND Patellofemoral AND Ligament]). Duplicates were removed from each search before title and abstract screening. Two independent reviewers examined the title and/or abstract of 611 studies in duplicate to determine study eligibility based on the inclusion and exclusion criteria. A third reviewer made the final decision for any disagreements. Two independent reviewers conducted full‐text reviews of 54 included studies in duplicate to determine final inclusion for the systematic review. A third reviewer made the final decision for any disagreements. This process yielded 12 studies for data extraction.

### Data extraction

Two authors independently conducted a thorough data extraction from the final 12 studies, which was reviewed by a third author. Only two were comparative studies on MPFL reconstruction comparing HT and QT autografts. Patient demographics, including age, gender, number of patients and knees, procedure type and choice of autograft were collected. Similarly, outcomes included in the study analysis were extracted. Since only two studies included comparison groups, a meta‐analysis of the results was not conducted. In comparative studies that included a standard‐of‐care group compared to a nonstandard‐of‐care group, only data from the standard‐of‐care group was extracted.

### Risk of bias assessment

The methodological index for nonrandomised studies (MINORS) criteria was used by two authors independently to assess the quality of included studies and determine the risk of bias [27]. The MINORS criteria apply as a 0 for a parameter that is not reported, a 1 for a parameter that is reported but inadequately, and a 2 for a complete parameter that is adequately reported [27]. For noncomparative studies, the maximum score was 16, with a score of 0–8, 9–14 and 15–16 equating to a poor, fair and high‐quality study, respectively [30]. For comparative studies, the maximum score was 24, with a score of 0–14, 15–22 and 23–24 equating to poor, fair and high quality, respectively [30].

### Statistical analysis

Quantitative syntheses and other statistical analyses were not conducted due to significant heterogeneity among the studies, with only two studies having comparison groups. The primary outcome and secondary outcomes were provided in ranges and table format, and meta‐analysis was not performed. For patient‐reported outcome measures (PROMs), the arithmetic mean and standard deviation were used as representative statistics and the mean difference was used to evaluate improvement after surgery.

## RESULTS

The literature search from each database resulted in 948 total articles. Three hundred and thirty‐seven duplicates were removed, leaving 611 studies for title and/or abstract screening. Five hundred and sixty‐seven studies were excluded based on inclusion and exclusion criteria, and 54 studies moved forward for full‐text screening. Forty‐two studies were further excluded after a thorough full‐text examination, yielding a final 12 articles for data extraction (Figure 1). Two of them were comparative studies with HT and QT autograft groups, while 10 studies either had a QT autograft group or an HT autograft group. These noncomparative studies were included due to a lack of a significant number of comparative studies available on this topic.

### Study demographics

Twelve studies that met the final inclusion criteria were included in this review. Seven studies used HT autograft, three studies used QT autograft, and two comparative studies had both HT and QT autograft patient groups. A summary of study demographics can be seen in Table 1. In this review, we did not perform a subgroup analysis between semitendinosus and gracilis autografts in the HT group. A total of 339 patients in this review underwent isolated MPFL reconstruction using HT autograft, while 139 patients received QT autograft. The mean age of the HT autograft group ranged from 19.6 to 25.9 years, and for the QT autograft group, it ranged from 19.4 to 26.2 years.

**Table 1:** Baseline patient demographics and study characteristics.

Reference	Study type	Comparison group	Mean age (years)	Minimum follow‐up (months)	Females (*n*)	Patients (*n*)	Autograft type	Femoral fixation type	Patellar fixation type
[1]	Retrospective case series	‐	23.4	24	22	38	Hamstring	Interference screw	2 suture anchors
[14]	Prospective comparative study	Gracilis tendon versus Semitendinosus autograft	24.6	24	19	28	Hamstring	Suture anchor	V‐shaped tunnel, Hi‐Fi sutures
[24]	Retrospective comparative study	Hamstring versus Quadriceps autograft	QT: 26.2 HT: 25.9	24	QT: 10 HT: 14	QT: 21 HT: 22	Quadriceps and Hamstring	Interference screw in both groups	Quadriceps: not applicable Hamstring: patellar tunnel, suture loop
[32]	Retrospective case series	‐	22	28	7	16	Quadriceps	Femoral with suture anchor	Not applicable
[23]	Retrospective comparative study	Hamstring versus Quadriceps autograft	QT: 20 HT: 20	24	QT: 15 HT: 15	QT: 32 HT: 32	Quadriceps and Hamstring	Interference screw in both groups	Quadriceps: not applicable Hamstring: patellar tunnel, suture anchor
[26]	Retrospective case series	‐	25	120	37	54	Hamstring	Femoral with interference screw	Patellar tunnel with suture loop
[22]	Prospective case series	‐	25.1	24	17	36	Quadriceps	Femoral with interference screw	Not applicable
[5]	Prospective case series	‐	19.4	24	29	34	Quadriceps	Femoral with interference screw	Not applicable
[7]	Retrospective cohort	MPFL reconstruction with anteromedialization versus isolated MPFL	19.6	24	10	14	Hamstring	Femoral with Endobutton	Patellar with suture anchor
[19]	Prospective randomised comparative trial	Two‐strand semitendinosus grafts versus four‐strand gracilis and semitendinosus grafts	25.8	36	26	38	Hamstring	Femoral with interference screw	Two semi‐patellar tunnels, suture loop
[33]	Prospective case series	‐	21	24	46	68	Hamstring	Femoral with interference screw	Patellar with suture anch ors
[11]	Prospective case series	‐	22.82	24	28	45	Hamstring	Femoral with interference screw	Two transverse tunnels, suture loop

Abbreviations: HT, hamstring tendon; MPFL, medial patellofemoral ligament; QT, quadriceps tendon.

### Risk of bias assessment using MINORS tool

The risk of bias assessment was conducted using the MINORS tool, which showed one study at high quality, 11 studies at fair quality, and none at poor quality (Table 2) [30].

**Table 2:** Risk of bias assessment using methodological index for nonrandomised studies tool.

S.no	Study	A clearly stated aim	Inclusion of consecutive patients	Prospective collection of data	Endpoints appropriate to the aim of the study	Unbiased assessment of the study endpoints	Follow‐up period appropriate to the aim of the study	Loss to follow‐up of less than 5%	Prospective calculation of the study size	An adequate control group	Contemporary groups	Baseline equivalence of groups	Adequate statistical analysis	Total	Quality of study assessment
1.	Basso et al.	2	2	1	2	2	2	2	0	—	—	—	—	13/16	Fair quality
2.	Marot et al.	2	2	2	2	2	2	0	1	—	—	—	—	13/16	Fair quality
3.	Sanguanjit et al.	2	1	0	2	2	2	2	1	2	1	2	2	19/24	Fair quality
4.	Vavalle et al.	2	2	1	2	2	2	2	0	—	—	—	—	13/16	Fair quality
5.	Runer et al.	2	1	0	2	2	2	1	1	2	1	2	2	18/24	Fair quality
6.	Shatrov et al.	2	2	1	2	2	2	0	0	—	—	—	—	11/16	Fair quality
7.	Peter et al.	2	2	2	2	1	2	2	2	—	—	—	—	15/16	High quality
8.	Fadel et al.	2	2	2	2	1	2	2	0	—	—	—	—	13/16	Fair quality
9.	Hashimoto et al.	2	2	1	2	2	2	1	0	—	—	—	—	12/16	Fair quality
10.	Niu et al.	2	2	2	2	2	2	0	2	—	—	—	—	14/16	Fair quality
11.	Zhang et al.	2	1	2	2	2	2	0	0	—	—	—	—	11/16	Fair quality
12.	Ibrahim et al.	2	1	1	2	1	2	2	0	—	—	—	—	11/16	Fair quality

### Re‐dislocation rate

Only one study, Shatrov et al., who used HT autograft, reported postoperative re‐dislocations with four patients needing revision surgery [26]. All other studies reported no patellar re‐dislocation in their patients.

### Functional outcomes

Kujala scores (anterior knee pain scales) from the final set of 12 studies are shown in Table 3. The mean improvement value was the difference between post‐ and preoperative mean functional outcome score, which formed a range from the lowest to the highest mean improvement from each study. Preoperative score was determined before MPFLR, and postoperative score was determined from the final follow‐up. All nine studies that reported pre‐ and postoperative mean Kujala scores had a significant mean improvement (HT group ranged from 26.5 to 41.4, QT group ranged from 6.6 to 53.0) [1, 5, 7, 11, 14, 19, 22, 32, 33]. Mean preoperative Kujala scores ranged from 50.2 to 68.4 for the HT autograft group and from 35.8 to 82.1 for the QT autograft group. Mean postoperative Kujala scores ranged from 82.9 to 94.9 for the HT group and from 88.4 to 94.8 for the QT group.

**Table 3:** Pre‐ and postoperative mean Kujala score in HT and QT groups (—, not reported).

Reference	Autograft type	Preoperative Kujala score (mean ± SD)	Postoperative Kujala score (mean ± SD)	Mean Improvement (mean ± SD)	Sample size	*p* value
[1]	Hamstring	57.2 ± 12.9	93.5 ± 6.9	36.3 ± 14.63	38	*p* < 0.0001
[14]	Hamstring	61.7 ± 14.9	92.1 ± 9	30.4 ± 17.41	28	*p* < 0.0001
[24]	Quadriceps and Hamstring	—	QT: 94.9 ± 4.1 HT: 94.2 ± 8.0	—	QT: 21 HT: 22	0.73
[32]	Quadriceps	35.8 ± 5.5	88.8 ± 4.3	53.0 ± 6.98	16	*p* < 0.001
[23]	Quadriceps and Hamstring	—	QT: 88.4 ± 5.0 HT: 89.4 ± 10.2	—	QT: 32 HT: 32	0.20
[26]	Hamstring	—	82.9 ± 15.3	—	54	—
[22]	Quadriceps	82.1 ± 12.5	88.7 ± 4.5	6.6 ± 13.29	36	0.01
[5]	Quadriceps	69.5 ± 4.6	94.8 ± 2.9	25.3 ± 5.44	34	0.01
[7]	Hamstring	68.4 ± 10.1	94.9 ± 5.9	26.5 ± 11.7	14	0.001
[19]	Hamstring	50.2 ± 5.5	91.6 ± 3.5	41.4 ± 6.52	38	0.000
[33]	Hamstring	57.53 ± 8.59	89.51 ± 3.9	31.98 ± 9.43	68	0.000
[11]	Hamstring	53.88	86.24	32.36	45	0.001

Abbreviations: HT, hamstring tendon; QT, quadriceps tendon.

Vavalle et al., Peter et al., Niu et al. and Zhang et al. provided preoperative and postoperative mean Lysholm scores from their patients, shown in Table 4 [19, 22, 32, 33]. All four studies from both the HT and QT autograft groups showed a significant mean improvement in pre‐ to postoperative Lysholm score (the HT group ranged from 41.7 to 45.84, the QT group ranged from 10.9 to 46.0). Mean preoperative Lysholm scores for the HT group ranged from 43.53 to 48.6, and for the QT group, ranged from 43.3 to 79.3. Mean postoperative Lysholm scores for the HT group ranged from 89.37 to 90.3, and for the QT group, ranged from 89.3 to 90.2.

**Table 4:** Preoperative and postoperative mean Lysholm score in HT and QT groups from a subset of four studies.

Reference	Autograft type	Preoperative Lysholm score (mean ± SD)	Postoperative Lysholm score (mean ± SD)	Mean improvement (mean ± SD)	Sample size	*p* value
[19]	Hamstring	48.6 ± 6.5	90.3 ± 5.1	41.7 ± 8.26	38	*p* < 0.00001
[33]	Hamstring	43.53 ± 10.20	89.37 ± 4.38	45.84 ± 11.1	68	—
[22]	Quadriceps	79.3 ± 16.1	90.2 ± 9.6	10.90 ± 18.74	36	*p* < 0.02
[32]	Quadriceps	43.3 ± 6.4	89.3 ± 3.1	46.0 ± 7.11	16	*p* < 0.001

### Return to sports (RTS)

Shatrov et al., Peter et al., Zhang et al., Sanguanjit et al. and Runer et al. provided postoperative mean Tegner activity scores, as shown in Table 5 [22, 23, 24, 26, 33]. In these five studies, mean postoperative Tegner activity scores for the HT group ranged from 4.0 to 7.6 and for the QT group from 5.5 to 6.0.

**Table 5:** Postoperative mean Tegner activity score from a subset of five studies.

Reference	Autograft type	Postoperative Tegner activity score (mean ± SD)	Sample size
[26]	Hamstring	4.0 ± 1.7	54
[14]	Hamstring	4.91	28
[33]	Hamstring	7.26 ± 0.78	68
[23]	Hamstring	4.6 ± 1.8	32
[23]	Quadriceps	5.5 ± 1.9	32
[22]	Quadriceps	6.0	36

Basso et al. (HT) allowed controlled sports 4 months after MPFLR and reported that patients had good clinical outcomes and returned to full activity in 6–12 months [1]. Sanguanjit et al. (QT + HT), Runer et al. (QT + HT), Peter et al. (QT), and Ibrahim et al. (HT) reported that patients were allowed to RTS 4–6 months after surgery [11, 22, 23, 24]. Fadel et al. (QT) reported that the gradual RTS was allowed 3 months postsurgery [5]. Hashimoto et al. (HT) and Niu et al. (HT) reported that patients were allowed to RTS 6 months postsurgery [7, 19]. Zhang et al. (HT) reported that individual and contact sports were allowed between 3 to 6 months following surgery [33]. Marot et al. (HT) and Shatrov et al. (HT) did not report a RTS time [14, 26]. All previously mentioned studies did not mention specifically how many patients returned to sports and to what degree or level. Only Vavalle et al. (QT) gave specific data on patients' RTS: five out of nine patients who played sports returned at the same preinjury level, one decreased in level, and three retired from playing their sport [32].

### Patellar tilt angle (PTA)

Basso et al., Fadel et al., Hashimoto et al. and Niu et al. provided both preoperative and postoperative mean PTA (Table 6) [1, 5, 7, 19]. All four studies showed significant decreases in PTA following surgery. The mean improvement, defined by the difference from pre‐ to postoperative value, with improvement being a decrease in PTA, for the HT group ranged from 12.6 to 16.3. The mean improvement in PTA for the one study from the QT group was 13.0.

**Table 6:** Change in patellar tilt angle from a subset of four studies.

Reference	Autograft type	Preoperative patellar tilt angle (mean ± SD)	Postoperative patellar tilt angle (mean ± SD)	Mean improvement (mean ± SD)	Sample size	*p* value
[1]	Hamstring	28.2 ± 8.7	13.8 ± 6	14.4 ± 10.57	38	<0.0001
[5]	Quadriceps	14.8 ± 5	1.8 ± 1.5	13.0 ± 5.22	34	<0.01
[7]	Hamstring	31.4 ± 7.2	18.8 ± 4.6	12.6 ± 8.54	14	<0.001
[19]	Hamstring	30.3 ± 7.7	14 ± 2.8	16.3 ± 8.19	38	<0.001

### ROM

Marot et al. (HT) reported no difference between pre‐ and postoperative knee ROM values [14]. Vavalle et al. (QT), Runer et al. (QT + HT), Shatrov et al. (HT), Peter et al. (QT), Fadel et al. (QT), Niu et al. (HT) and Ibrahim et al. (HT) all reported that all patients had a full ROM postoperatively [5, 11, 19, 22, 23, 26, 32]. Zhang et al. (HT) specifically reported a mean preoperative ROM of 121.44 degrees and a mean postoperative ROM of 130.93 degrees at final follow‐up [33]. Sanguanjit et al. (QT + HT) reported at final follow‐up a mean postoperative ROM of 140 degrees for the QT autograft group and 138 degrees for the HT autograft group [24]. Basso et al. and Hashimoto et al. did not report on ROM [1, 7].

### VAS for pain

Five studies reported data from their patient cohort using the VAS to measure pain [1, 5, 14, 22, 23]. Basso et al. (HT) reported a mean preoperative VAS score of 4.4 and a mean postoperative VAS score of 1.3, showing a significant decrease (*p* < 0.0001) [1]. Marot et al. (HT) reported a mean VAS decrease of −4.3 from preoperative to final follow‐up [14]. Runer et al. (QT + HT) reported at final follow‐up a mean VAS of 1.1 for the QT autograft group and a VAS of 1.9 for the HT autograft group [23]. Peter et al. (QT) reported a mean preoperative VAS score of 2.5 and a mean postoperative VAS score of 1 at final follow‐up, with a significant decrease (*p* < 0.01) [22]. Fadel et al. (QT) reported a mean preoperative VAS score of 5 and a mean postoperative VAS score of 1, also with a significant decrease (*p* < 0.0001) [5]. From these studies, both hamstring and quadriceps autograft groups had significant decreases in mean VAS score at final follow‐up.

### Radiographic analysis

Three HT group studies and one QT group study reported data on PTA [1, 5, 7, 19]. Basso et al. (HT), Niu et al. (HT), Hashimoto et al. (HT), and Fadel et al. (QT) all reported that the PTA decreased significantly from pre‐ to postoperation, as shown in Table 6. Shatrov et al. (HT) reported patients who had no signs of patellofemoral arthritis (PFA) prior to surgery but developed PFA on postoperative imaging. At the final follow‐up, 33 patients (66%) showed no arthritis, 15 patients (30%) showed Iwano stage I PFA, and two patients (4%) had Iwano stage 2 PFA. Five patients showed signs of PFA in the contralateral knee without patellofemoral instability that developed during their study [26].

### Complications

Basso et al. (HT) reported that three patients required new surgery: one for scar revision and two for removal of femoral interference screw due to cyst formation [1]. Marot et al. (HT) reported one case of subjective patellar instability [14]. Shatrov et al. (HT) reported one patient with postoperative haematoma, one patient with ACL reconstruction, and one patient with arthroscopic arthrolysis [26]. Peter et al. (QT) reported one patient with a positive postoperative apprehension test and one patient with an intraoperative complication‐ the graft was cut too short, requiring a second strip of tendon [22]. Fadel et al. (QT) reported that at 3 months postop, one patient had persistent limited flexion and two patients had persistent quadriceps atrophy, which needed electrical stimulation [5]. Zhang et al. (HT) reported two obese patients with a delayed union of incision, three patients with skin hypaesthesia on the anterior knee, and two patients with patellar clicking at 30° knee flexion [33]. Niu et al. (HT), Sanguanjit et al. (QT + HT), and Ibrahim et al. (HT) reported that patients had no complications [11, 19, 24]. Hashimoto et al. did not clarify information regarding complications [7]. Only Runer et al. (QT + HT) reported data on donor site morbidity, and their study reported significantly more patients (59.4%) treated with HT autograft had a sensitivity deficit in the lower leg compared to the QT autograft patient group (3.1%, *p* = 0.001) [23].

## DISCUSSION

The primary outcome of this systematic review was the patella re‐dislocation rate, and 11 out of the 12 included articles reported no instances of re‐dislocation. One study using gracilis tendon autograft (HT group) reported a 7.4% rate of recurrent postoperative instability that required revision surgery [26]. This finding shows no significant difference in re‐dislocation rate between HT and QT autografts for MPFLR. This result reflects findings from another systematic review of 11 studies using the superficial ‘swing‐down’ QT autograft for MPFL reconstruction, which also found no cases of patellar re‐dislocation [20].

Regarding the autograft options for MPFLR, the HT and QT autografts are commonly used [15]. A recent technical note by Camanho et al. mentioned the patellar tendon autograft as an option for MPFL reconstruction [3]. In this systematic review of 12 studies, seven used HT autografts, three used QT autografts, and two comparative studies included both HT and QT autografts. Migliorini et al. noted that in their review of 149 patients undergoing MPFL reconstruction with quadriceps pedicled strip autografts, five patients had persistent joint instability [18]. However, none experienced re‐dislocation or required reoperation. In a systematic review of synthetic grafts for MPFL reconstruction, 2.5% (5/199) of patients had re‐dislocation, and less than 1% had undergone revision surgery [16]. However, in Migliorini et al., their comparative study found a significant decrease in re‐dislocation rate and revision surgery in the semitendinosus group compared to the gracilis group [17]. They also noted that while the semitendinosus tendon is stronger than the gracilis in terms of tensile strength, the gracilis has a tendon stiffness value that is closer to that of the native MPFL. While a slight increase in strength is noted with the semitendinosus tendon, this must be weighed against the persistent loss of hamstring strength incurred from its harvest compared to the gracilis tendon [25].

Regarding PROMs, both the HT and QT autograft groups showed significant improvement in postoperative Kujala scores compared to preoperative scores. Similarly, for Lysholm scores, both groups demonstrated significant improvement in postoperative scores. Runer et al. compared the gracilis tendon autograft and QT autograft for MPFL reconstruction and found similar results in terms of Kujala and Lysholm scores. In both groups, there was a significant increase in the postoperative Tegner activity score, but no significant difference between the groups. However, Runer et al.‘s comparative study observed a significant increase in the Tegner activity level in the QT group compared to the HT group (*p* = 0.027), though the difference was within the minimal clinically significant range [23]. The VAS for pain significantly improved after surgery in both HT and QT groups. The main limitation is that these outcome measuring tools are not specific to patellofemoral instability cases, and Smith et al. mentioned this limitation in their article [28]. To overcome this limitation, Hiemstra et al. created the Banff Patella Instability Instrument (BPII), a validated 32‐item questionnaire to evaluate quality of life outcomes specific to patellofemoral instability cases [8]. Later, after modification, BPII 2.0 came with 23 items, a valid and reliable outcome tool to assess patella instability cases, both surgically and nonsurgically treated [13]. Another validated PROM tool specific to patellofemoral instability cases is the Norwich Patellar Instability Score, which is a 19‐item questionnaire [29]. However, none of the studies included in our review used these specific outcome tools for assessment.

RTS after surgery varied across studies, ranging from 3 to 12 months. A gradual RTS after 6 months seems the safest approach. Different fixation techniques were used in the HT group, including patellar suture anchors and femoral‐side fixations with either interference screws or suture anchors (Table 1). In the QT group, all fixations were performed on the femoral side. This is one of the main purported advantages of using the QT, as it could eliminate patellar fracture as a potential complication of this procedure. Interestingly, patellar fracture as a complication was not reported in any of the studies assessing the use of HT. In a recent systematic review of the complications of MPFL reconstruction, the incidence of patellar fracture was found to be 0%–8.3%, primarily where patellar fixation involved a full‐length transverse tunnel or a two‐tunnel technique [12]. Of the nine studies using HT in our review, five used full‐length patellar tunnels in a total of 219 patients [11, 14, 19, 24, 26].

Regarding PTA, both groups showed a significant change in postoperative tilt compared to preoperative values. In the HT group, Zhang et al. observed anterior knee skin hypaesthesia in three patients, while in the QT group, Fadel et al. reported that two patients experienced persistent quadriceps atrophy and required electrical stimulation [5, 33]. Regarding donor site morbidity, Runer et al., in their comparative study, found that the HT group had a significantly greater sensory deficit in the lower limb compared to the QT group [23]. Based on this study, donor site morbidity appears to be lower in the QT group, making it a more promising option. None of the included studies measured the strength of the HT or QT following MPFLR.

### Limitations

This review has some limitations. The quality of the studies included is generally low, with most of them providing level 3 and 4 evidence. Notable heterogeneity was seen among the studies, and only two recent papers included a comparison group. We did not perform a meta‐analysis of our findings, given this lack of a comparison group in the overwhelming majority of the studies. We only compared HT autograft to QT autograft and did not include other graft options, such as the peroneus longus, adductor magnus, patellar tendon, allografts or synthetic grafts. Given the significant heterogeneity of the available studies and a previous systematic review indicating the superiority of HT and QT autografts compared to other graft options, we elected to limit the review to HT and QT autografts [15]. Additionally, other bony factors that contribute to graft failure or postoperative instability were not considered. The secondary outcome parameters, like the Kujala score, Lysholm and Tegner activity scores, are not specific and validated for patellofemoral instability cases. Nevertheless, to the best of our knowledge, this is the first systematic review focused exclusively on two commonly used autografts (HT vs. QT) for MPFLR with a minimum 24‐month follow‐up.

## CONCLUSIONS

There is no difference in re‐dislocation rate or PROMs between isolated MPFL reconstruction done with the HT compared to QT autograft. Major limitations of this systematic review include a lack of strong outcome measures specific to patients with patellofemoral instability, along with few comparative studies. Future studies should focus on using validated PROMs for patellofemoral instability patients.

## AUTHOR CONTRIBUTIONS

All authors contributed to the study conception and design. Material preparation, data collection and analysis were performed by Ehson Maleki, Ali Ahmadi Pirshahid, Lee R. Benaroch and Karthick Rangasamy. The first draft of the manuscript was written by Ehson Maleki and all authors commented on previous versions of the manuscript. All authors read and approved the final manuscript.

## CONFLICT OF INTEREST STATEMENT

The authors declare no conflicts of interest.

## ETHICS STATEMENT

As this is a systematic review of the exisitng literature, approval by Institute Ethics committee was deemed unnecessary.

## Data Availability

Data sharing is not applicable to this article as no datasets were generated or analysed during the current study.
